# Eighteen-Year Incidence, Health Outcomes and Costs Associated With Diabetic Ketoacidosis at Diagnosis of Type 1 Diabetes in Children in NSW, Australia

**DOI:** 10.1155/pedi/2550952

**Published:** 2025-07-21

**Authors:** Kirstine J. Bell, Samantha J. Lain, Lindsay Stevens, Maria E. Craig, Kim C. Donaghue, Natasha Nassar

**Affiliations:** ^1^Charles Perkins Centre, The University of Sydney, Sydney, New South Wales, Australia; ^2^Children's Hospital at Westmead Clinical School, The University of Sydney, Sydney, New South Wales, Australia; ^3^Institute of Endocrinology and Diabetes, The Children's Hospital at Westmead, Sydney, New South Wales, Australia; ^4^Discipline of Child and Adolescent Health, University of Sydney, Sydney, New South Wales, Australia; ^5^Discipline of Paediatrics and Child Health, School of Clinical Medicine, UNSW Health, University of New South Wales, Sydney, New South Wales, Australia

**Keywords:** children, diabetic ketoacidosis, type 1 diabetes

## Abstract

**Aim:** Diabetic ketoacidosis (DKA) is a life-threatening complication of type 1 diabetes (T1D). We determined the incidence, trends, cost and characteristics of children with and without DKA at T1D diagnosis and association with DKA readmissions.

**Methods:** Children aged <16 years with T1D and residing in New South Wales, Australia, were identified from population-based hospital records (Admitted Patient Data Collection; APDC) for 2002–2019. Diagnoses of T1D and DKA were identified using ICD10 codes. Costs were determined using the ‘Australian Refined-Diagnosis Related Group' (AR-DRG) code multiplied by the cost weight and National Efficient Price for the admission year. Associations were assessed using Chi-squared analyses and multivariate regression.

**Results:** A total of 5832 children with T1D were identified, and 36% had DKA at diagnosis. The proportion with DKA at diagnosis was 34.4% in 2002–2003 and 41.0% in 2018–2019, with limited evidence to support a meaningful change over time (Cochrane-Armitage test-for-trend, *p*=0.062). DKA at diagnosis was associated with age <2 years, lower socio-economic status (SES) and rural/regional areas. DKA at diagnosis was also associated with an increased risk of readmission(s) for DKA (odds ratio [OR]: 1.35 [95% confidence interval [CI] 1.23, 1.49]). DKA doubled the costs/person, considering all available follow-up ($20,571 [interquartile range: $10,825, $37,845] vs. $9743 [$4980, $18,287]).

**Conclusion:** DKA at diagnosis of T1D is a common and expensive health issue in Australia, with the rate of DKA at diagnosis not improving over the last two decades. Effective strategies are needed to improve health outcomes and reduce the economic burden.

## 1. Introduction

Type 1 diabetes (T1D) is an autoimmune condition resulting from the immune-mediated destruction of the insulin-producing pancreatic beta-cells. Three children are diagnosed every day in Australia, with a prevalence of 144 per 100,000 children between 2002 and 2017 and peak incidence between 10 and 14 years of age [1]. Management of T1D requires life-long insulin therapy for survival, with the goal of maintaining blood glucose levels within the narrow limits of normoglycaemia. Suboptimal glycaemia is closely associated with serious acute and chronic diabetes complications, such as severe hypoglycaemia, recurrent diabetic ketoacidosis (DKA), diabetic eye disease (retinopathy, macular oedema, blindness), nephropathy/chronic kidney disease, neuropathy (which can lead to amputation), reduced lifespan and significant psychological morbidity [2].

DKA is a life-threatening complication of T1D, resulting from excess production of ketones due to absolute or relative insulin deficiency [3]. DKA at the time of T1D diagnosis has been associated with immediate and long-term outcomes, including cognitive impairment [4, 5], recurrent hospital admissions for DKA [6] and consistently higher HbA1c levels, independent of demographic or socio-economic factors [7, 8]. Treatment of DKA requires emergency medical care, often with admission to intensive care, necessitating expensive high-level medical care and causing significant post-traumatic stress for families [9]. Left untreated, DKA causes coma and death [6].

Given the potential health implications of DKA at diagnosis of T1D, this study therefore aims to determine the incidence, trends and characteristics of Australian children with and without DKA at diagnosis of T1D. Secondary aims are to determine the health systems costs associated with DKA at diagnosis (as incurred by the Australian Government) and explore the association with the risk of recurrent DKA.

## 2. Methods

### 2.1. Population

All children aged less than 16 years identified as having T1D in a hospital/day stay record between 1 January 2002 and 31 December 2019 in NSW, Australia, were included in this study. NSW is the most populous state in Australia, with ~8.33 million residents (31% of Australia's population). Children were excluded if they did not reside in NSW or if their diabetes diagnosis was secondary to another condition such as cystic fibrosis, thalassaemia major, neonatal diabetes or pancreatectomy for neonatal hyperinsulinism.

Data were sourced from the hospital data through the NSW Admitted Patient Data Collection (APDC) from 1 January 2002–31 December 2019 (complete available dataset at time of study). The APDC captures all episodes of care in public and private hospitals, multipurpose services and day-stay procedure centres in NSW. It includes information on demographics, timing of admission, clinical diagnoses and procedures performed during the episode of care. These are coded using the International Classification of Diseases version 10-Australian Modification (ICD10-AM) and Australian Classification of Healthcare Interventions (ACHI).

A diagnosis of T1D was identified in the APDC dataset using the ICD10-AM code of E10 (E10.0–E10.9), and DKA was identified using the ICD10-AM code of E10.11–E10.12, E10.15–E10.16. These codes have previously been used in the literature [6] and by the Australian Institute of Health and Welfare (AIHW) for population reports [10]. The date of T1D diagnosis was assumed to be the date of admission with the first record of the T1D ICD10-AM code, given there was no prior coding for T1D in the previous decade. This assumption, overall data completeness, was previously validated against the explicit date of diagnosis reported in the Australasian Paediatric Endocrine Group (APEG) NSW Childhood Diabetes Register available for the period of data overlap with APDC between 2001 and 2009. In this validation, 90% of children had a matched first presentation with a T1D ICD10-AM code in APDC within 30 days before or after the date of diagnosis in the APEG register (unpublished data). Children were followed until the end of the study on 31 December 2019 (maximum age at end of study: 32 years of age), with a median duration of follow-up of 9 years (Q1, Q3 5, 14).

### 2.2. Population Characteristics

Sex, age at diagnosis, year of diagnosis and statistical local areas (SLAs) were ascertained from the APDC. Age at diagnosis was categorised into <2, 2–4, 5–9 or 10–15 years inclusive. Socio-economic status (SES) was determined based on the Australian Bureau of Statistics Socio-Economic Index for Relative Disadvantage for each SLA of residence. SES was categorised into quintiles from most disadvantaged (Q1) to most advantaged (Q5) [11]. Remoteness of residence was categorised as either major cities or regional/remote and was determined using the Accessibility/Remoteness Index of Australia (ARIA) for each SLA [12].

### 2.3. Health System Costs

Costs of admissions with and without DKA at T1D diagnosis were explored using a government health system perspective, based on prespecified activity-based funding for public hospitals by the Australian Government, and allocated reimbursement costs. Costs were estimated from APDC data using the Australian Refined Diagnosis Related Group (AR-DRG) code (Version 6) assigned for each admission. The AR-DRG code is assigned by a clinical coder using a complex algorithm based on the patient's diagnoses and procedures, and other factors such as age, sex and length of stay recorded in each episode of care. Each AR-DRG is assigned a relative weight by the Australian Independent Health and Aged Care Pricing Authority, which reflects the average resources required to treat patients in that group [13] (2010 #1182). This cost weight was then multiplied by the National Efficient Price for the admission year to determine the cost per admission [14, 15].

### 2.4. Statistical Analysis

Descriptive statistics of characteristics of children and admissions are reported as mean and standard deviation (SD) for normally distributed continuous variables and median and interquartile (Q1 and Q3) for non-normally distributed continuous variables. Differences between groups were evaluated using *χ*^2^ tests or Brunner–Munzel tests, and trends in proportion of DKA events (hereafter called 'DKA rate') over time were examined using the Cochrane–Armitage test for trend. Associations between explanatory variables and DKA at diagnosis were analysed using multivariate logistic regression analysis. We first examined the crude association between each characteristic and the risk of DKA at diagnosis. The multivariate logistic models were then adjusted for all sociodemographic variables, age at diagnosis and year of diagnosis. Cox regression models were used to account for the different follow-up durations for children in the cohort. The Kaplan–Meier survival probability with 95% confidence interval (CI) was calculated for the risk of readmission for DKA following diagnosis for children with and without DKA at diagnosis. Adjusted hazard ratios (aHRs) with 95% CIs were calculated, adjusted for characteristics at diagnosis. For *χ*^2^ tests, Brunner–Munzel test and Cochrane–Armitage test for trend, *p*  < 0.05 were considered statistically significant, while Hazard and odds ratios (ORs) were significant when 95% CIs did not cross 1. All analyses were performed using SAS, version 9.4 software (SAS Institute). This study was approved by the NSW Population Health Services Research Ethics Committee (2019/ETH01580).

## 3. Results

**Figure 1:**
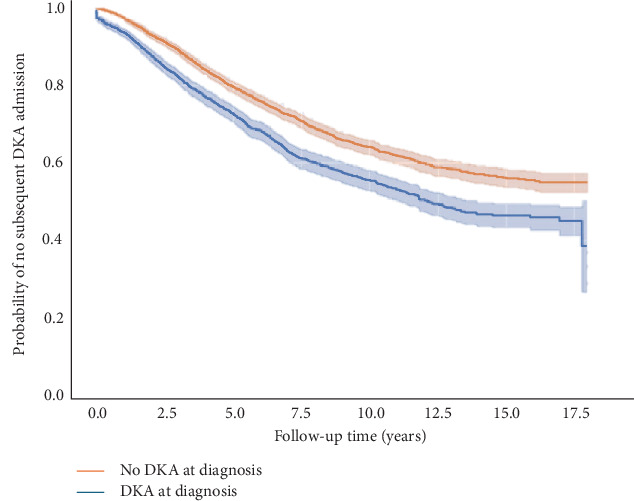
Trend in proportion of children presenting with diabetic ketoacidosis (DKA) at diagnosis of type 1 diabetes in NSW between 2002 and 2019. Test for trend 0.062.

A total of 5832 children were identified as being diagnosed with T1D in NSW between 2002 and 2019 in the APDC dataset. Of these, 36% (*n* = 2064) had DKA at diagnosis. The proportion with DKA at diagnosis was 34.4% (95% CI: 30.5%, 38.3%) in 2002–2003 and 41.0% in 2018–2019 (95% CI: 37.4%, 44.7%) with limited evidence to support a meaningful change over time (Cochran–Armitage test for trend over time; *p*=0.062; Figure 1).

There were no differences in the rate of DKA at diagnosis by sex. However, a younger age at diagnosis was associated with an increased risk of DKA at diagnosis. This was driven by the youngest children, aged <2 years of age, where 64% experienced DKA at diagnosis. The odds of a child <2 years old experiencing DKA at diagnosis were threefold higher than that of a 10–15 year old child (adjusted OR [aOR]: 3.19 [2.42, 4.22]). SES was also associated with DKA at diagnosis, with children living in the most disadvantaged communities having the greatest risk (aOR: 1.38 [1.16, 1.64]) (Table 1).

Most children with newly diagnosed T1D, regardless of DKA, presented to the emergency department (DKA: 90% vs. No DKA: 85%; *p* < 0.001), but those with DKA had a longer median length of stay (5 days, Interquartile range [Q1, Q3] 2, 7 days) vs. 4 days (Q1, Q3 2, 6 days; *p* < 0.001) and a higher proportion required an intensive care admission (19% vs. 1%; *p* < 0.001) and transfer between facilities (28% vs. 8%; *p* < 0.001; Table 2).

**Figure 2:**
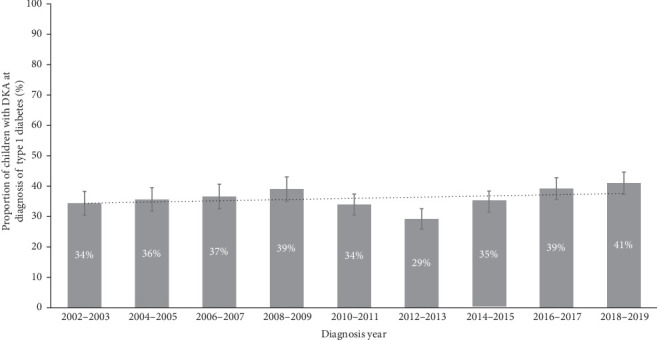
Kaplan–Meier survival curve for time to first diabetic ketoacidosis (DKA) admission following diagnosis with type 1 diabetes in children in NSW, Australia between 2002 and 2019.

Children who presented with DKA at diagnosis were more likely to have recurrent DKA (41% vs. 34%, aHR: 1.35 [1.23, 1.49]; Table 3). This effect was sustained and seen after up to 17.5 years after follow-up (Figure 2). Of note, 60% of those with DKA at diagnosis were readmitted for DKA within 18 years of diagnosis compared to 40% of those without DKA at diagnosis. Living in a lower SES area was associated with an increased risk of readmission for DKA (aHR: 1.73 [1.48–2.02]; Table 3). Being female and living in a regional/rural area were both associated with a slightly higher risk of readmission with DKA (OR: 1.25 [1.13, 1.37]; OR: 1.14 [1.03, 1.26]; respectively).

Children aged <2 years were most at risk of recurrent DKA, with 44% of children with DKA at diagnosis having had a recurrent event (*n* = 65) compared with 28% of children without DKA at diagnosis (*n* = 24; data not shown). However, younger age at diagnosis was not associated with recurrent DKA after taking into account other risk factors in the model (aHR: 1.19 [0.96, 1.49]). Approximately 30% of children with DKA at diagnosis required another hospital admission within 6 months of diagnosis, and almost half (47%) within the first year. In children without DKA at diagnosis, 25% and 42% experienced another hospital admission in the same timeframes (Figure 2).

The total cost to the Australian Government health system for admissions for T1D at the time of diagnosis over the 18-year period was AUD $14,084,142 for the 2097 youth with DKA at diagnosis vs $19,688,144 for the 3716 children without DKA at diagnosis (Table 4). The total costs to the government for all admissions, using all available follow-up, were AUD $106,761,027 for those who presented with DKA either at diagnosis or during any readmission vs. AUD $44,201,477 for those who never experienced DKA during the follow-up period. The median cost per individual across all admissions was also more than twice as high for individuals with DKA compared to those who never had DKA (AUD $20,571 ($10,825, $37,845) vs. $9743 ($4980, $18,287; Table 5).

## 4. Discussion

Over the past 18 years, on average, one in three children had DKA at diagnosis. The proportion with DKA at diagnosis was 34.4% in 2002–2003 and 41.0% in 2018–2019, with no change in rates over time (*p*=0.062). One in five children with DKA required an intensive care admission. DKA at diagnosis was also associated with an increased and sustained risk of readmission(s) for DKA. Very young children (aged <2 years) and those in lower SES or rural/regional areas were at higher risk of DKA at diagnosis. Future risk of DKA was 50% higher for those with DKA at diagnosis and was more likely among females, those from disadvantaged and regional areas. While the initial cost to the health system of DKA at diagnosis versus no DKA was similar, the median costs per person across all admissions were double for those who experienced DKA compared to those who did not.

Children under 2 years of age with T1D represent a very small proportion of children diagnosed with T1D (~4% of children in the present study) but are at a greater risk of DKA at diagnosis of T1D compared with older children [16]. This is potentially due to greater difficulty in recognising the signs and symptoms in very young children and the relative rarity of the condition in this age group, which can delay diagnosis and thereby increase the risk of DKA [17]. Furthermore, it has been suggested that T1D onset in very early childhood may be a more aggressive form of the condition [18, 19], which could contribute to the greater risk of DKA at diagnosis.

Our findings build on the evidence on DKA at diagnosis of T1D from Australia. Compared with an earlier study using the same NSW data source, our results report an increase in DKA at 41% in 2019 compared with the previous rate of 33% (2001–2013) [6]. Authors also noted that DKA at diagnosis was associated with a need for intensive care, a longer hospital stay, and subsequent readmission for DKA [6]. A state-wide hospital audit in Queensland revealed 45% of children were presenting with DKA on average, with rates as high as 80% in regional areas [20]. In Victoria, 20%–41% of children presented with DKA at diagnosis between 2017 and 2020 [20, 21]. These Australian rates of DKA are also consistent with international reports of DKA rates between 20% and 50% in New Zealand [16, 22], Europe, North America and Asia [23].

The strengths of this study are the use of the large, population-based data set that covers all of NSW—the most populous state in Australia. It incorporates and extends on earlier reports and, to our knowledge, now captures the longest period of time examining DKA at diagnosis for T1D in Australia. Furthermore, this is also the first study to have explored the government expenditure associated with admissions for DKA at diagnosis in Australia, which is needed to understand the scale of the economic burden and the potential savings for future cost-effectiveness evaluations of DKA prevention initiatives.

The study has several limitations. The diagnosis admission was assumed to be the first presentation coded with T1D, which was shown to be appropriate in a previous validation study, with at least 90% agreement. There is a chance our dataset includes a small number of children with type 2 diabetes who were clinically misdiagnosed or miscoded as having T1D. As a NSW-specific dataset, it does not capture children moving in and out of state, which may have impacted accurate identification of the diagnosis, admission or follow-up for subsequent readmissions. Furthermore, the APDC does not include Information on risk and protective factors associated with DKA at diagnosis identified in the literature, such as ethnicity and family history of T1D [24] and health outcome data, such as DKA severity or measures of immediate or long-term glycaemic control. Finally, the costs estimated for DKA may be underestimated as AR-DRGs are based on 'government-funded resource allocation costs' and may not be a sensitive instrument for measuring direct, hospital-level, healthcare expenditure associated with DKA, especially as there are only two AR-DRG codes for diabetes admissions. As such, the hospital resource utilisation costs for DKA may differ from the government-borne costs presented in this analysis.

Given the high incidence of DKA at diagnosis and associated costs, DKA prevention strategies are needed to improve health outcomes and reduce healthcare expenditure. Given the rate of DKA at diagnosis, with almost half of the cases experiencing DKA in more recent years, universal prevention initiatives are warranted rather than targeting specific populations (e.g., regional/rural communities). Public awareness campaigns to increase awareness of the signs and symptoms of T1D to reduce rates of DKA at diagnosis have had minimal impact or resulted in short-term awareness but lacked sustained outcomes [25, 26]. A systematic review and meta-analysis showed DKA rates pre- and post-campaign were reduced by 7.2%, whereas comparison between areas with and without a campaign differed by 35.7% [27]. Alternatively, general population screening for pre-symptomatic T1D in children has been shown to reduce the rate of DKA at diagnosis to less than 5% in large, international cohort studies [28] and may be cost-effective in areas with a high prevalence of DKA at diagnosis [29]. As a result, routine, population-wide screening for children has been recommended in international guidelines [23]. Early detection and education could eliminate the need for a hospital admission at the time of clinical diagnosis, traditionally required for glycaemic stabilisation and treatment of complications (e.g., DKA), intensive education and insulin initiation and titration. If successful, the cost of a national screening programme could be off-set by the avoidance of healthcare costs, not just for admissions with DKA at diagnosis, but potentially the vast majority of all diagnosis hospital admissions (combined total cost of over $33m over the past 18 years in NSW alone in present study).

In conclusion, DKA at and following diagnosis of T1D is a common and expensive health issue in Australia, and rates are not improving over time. Effective public health strategies are needed to reduce the economic burden and improve immediate and long-term health outcomes for Australian children.

## Figures and Tables

**Table 1:** Characteristics of children with and without DKA at diagnosis of type 1 diabetes.

Characteristics	DKA during diagnosis admission, *n* (%)	No DKA during diagnosis admission, *n* (%)	Univariate odds ratio (95% CI)	Multivariate odds ratio (95% CI)
Total	2097 (36)	3735 (64)	—	—
Characteristics at diagnosis				
Sex				
Male	1047 (50)	1944 (52)	—	—
Female	1050 (50)	1790 (48)	1.09 (0.98, 1.21)	1.11 (1.00, 1.24)
Age at diagnosis (inclusive)				
0–1	148 (7)	85 (2)	3.12 (2.37, 4.13)	3.19 (2.42, 4.22)
2–4	321 (15)	587 (16)	0.98 (0.84, 1.15)	0.98 (0.83, 1.14)
5–9	644 (31)	1297 (35)	0.89 (0.79, 1.01)	0.88 (0.78, 1.00)
10–15	984 (47)	1766 (47)	—	—
Socio-economic status				
1—most disadvantaged	453 (21)	710 (19)	1.41 (1.19, 1.66)	1.38 (1.16, 1.64)
2–4	1246 (60)	2148 (58)	1.28 (1.11, 1.47)	1.25 (1.08, 1.45)
5—least disadvantaged	391 (19)	862 (23)	—	—
Remoteness				
Metropolitan	1400 (67)	2590 (70)	—	—
Regional/rural	683 (33)	1124 (30)	1.12 (1.00, 1.26)	1.07 (0.95, 1.21)
Year of admission^a^			1.01 (1.00, 1.02)	1.01 (1.00, 1.02)

^a^Fit as a continuous variable centred on 2011.

**Table 2:** Characteristics of admissions for children with a new diagnosis of type 1 diabetes, by DKA at diagnosis status.

Characteristics	DKA during diagnosis admission, *n* (%)	No DKA during diagnosis admission, *n* (%)	*p*-Value^a^
Presented to ED			<0.001
ED involved	1894 (90)	3180 (85)	
Other or unknown	203 (10)	555 (15)	
Hospital type at diagnosis			<0.001
Principal referral^a^	602 (29)	1210 (32)	
Paediatric specialist	626 (30)	1202 (32)	
Private	9 (0)	50 (1)	
Other	860 (41)	1273 (34)	
Length of stay			<0.001
Length of stay (days), (median [Q1, Q3])	5 (2, 7)	4 (2, 6)	
ICU status (>0 h)			<0.001
Yes	408 (19)	22 (1)	
No	1689 (81)	3713 (99)	
Transferred			<0.001
Yes	579 (28)	291 (8)	
No	1518 (72)	3444 (92)	

^a^Formal term for Australian public hospitals with broad services and specialist facilities that accept referrals from lower level hospitals for complex cases.

**Table 3:** Survival model for time to first readmission for DKA in children with type 1 diabetes.

Characteristics	DKA on readmission, *n* (%)	No DKA during any readmission OR no readmission, *n* (%)	Univariate hazard ratio (95% CI)	Multivariate hazard ratio (95% CI)
Total	1829 (31)	4000 (69)	—	—
Years to first DKA				
Median (Q1, Q3)	3.82 (1.86, 6.54)	—	—	—
DKA at diagnosis				
Yes	752 (41)	1344 (34)	1.41 (1.28, 1.54)	1.35 (1.23, 1.49)
No	1077 (59)	2656 (66)	—	—
Patient characteristics, at diagnosis				
Sex			—	—
Male	861 (47)	2128 (53)	—	—
Female	967 (53)	1872 (47)	1.25 (1.14, 1.37)	1.24 (1.13, 1.36)
Age at diagnosis (inclusive)				
0–1	89 (5)	144 (4)	1.27 (1.02, 1.58)	1.19 (0.96, 1.49)
2–4	279 (15)	629 (16)	0.95 (0.83, 1.09)	0.94 (0.82, 1.08)
5–9	615 (34)	1323 (33)	1.02 (0.92, 1.13)	1.00 (0.90, 1.11)
10–15	846 (46)	1904 (48)	—	—
Socio-economic status				
1—most disadvantaged	423 (23)	739 (19)	1.84 (1.59, 2.14)	1.73 (1.48, 2.02)
2–4	1115 (61)	2279 (57)	1.54 (1.35, 1.75)	1.44 (1.25, 1.64)
5—least disadvantaged	286 (16)	965 (24)	—	—
Remoteness				
Metropolitan	1177 (65)	2810 (71)	—	—
Regional/rural	643 (35)	1164 (29)	1.25 (1.13, 1.37)	1.14 (1.03, 1.26)

**Table 4:** Costs of admissions for a new diagnosis of type 1 diabetes in children, by DKA at diagnosis status.

Characteristics	DKA during diagnosis admission	No DKA during diagnosis admission	*p*-Value^b^
Costs, totals	*n* (%)	*n* (%)	—
Persons	2097 (36)	3716 (64)	—
Cost, AUD	14,084,142	19,688,144	—
Costs, per person	Median (Q1, Q3)	Median (Q1,Q3)	—
Cost, AUD	4822 (4795, 9525)	4795 (4795, 4843)	<0.001
Admissions by diabetes-related AR-DRG code^a^			
K60B: DIABETES−CSCC	2552 (93)	3651 (92)	—
K60A: DIABETES + CSCC	117 (4)	28 (1)	—

Abbreviation: CSCC, catastrophic or severe complication and/or comorbidity.

^a^Only diabetes-related codes shown, so percentages do not add up to 100%.

^b^Brunner–Munzel test.

**Table 5:** Costs of all admissions, for any reason, for children with type 1 diabetes, using all available follow-up.

Costs	DKA at diagnosis or any readmission	No DKA ever OR no readmission	*p*-Value^a^
Costs, totals	*n* (%)	*n* (%)	—
Persons	3174 (55)	2642 (45)	—
Admissions	19,554 (70)	8439 (30)	—
Cost, AUD	106,761,027	44,201,477	—
Costs, per person	Median (Q1,Q3)	Median (Q1,Q3)	—
Admissions	4 (2, 7)	2 (1, 4)	<0.001
Length of stay, total	10 (6, 18)	6 (3, 9)	<0.001
Cost, AUD	20,571 (10,825, 37,845)	9743 (4980, 18,287)	<0.001
Costs, by top 5 AR-DRG codes			
K60B: Diabetes—CSCC	12844 (61)	4507 (52)	—
G67B: OESPHS, GASTR—CSCC	543 (3)	255 (3)	—
K60A: Diabetes + CSCC	656 (3)	38	—
G70B: Other digestive Sys Diag—CSCC	347 (2)	135 (2)	—
Z64B: OTH FCTR INFL health status, SD	223 (1)	202 (2)	—

Abbreviation: CSCC, catastrophic or severe complication and/or comorbidity.

^a^Brunner–Munzel test.

## Data Availability

The data that support the findings of this study are available from the NSW Ministry of Health, but restrictions apply to data access and availability, which require project-specific protocols, data custodian and ethics approval for use, and therefore are not publicly available.
